# Prion Folding Sends a Death Signal in Fungus

**DOI:** 10.1371/journal.pbio.1002058

**Published:** 2015-02-11

**Authors:** Richard Robinson

**Affiliations:** Freelance Science Writer, Sherborn, Massachusetts, United States of America

Prions are kind of like Batman—they have a bad reputation, but they can also serve a number of good purposes. While prion domains—folded regions of proteins that can induce similar folding in other susceptible proteins—are responsible for the human prion diseases and may be involved in other neurodegenerative diseases as well, they have also been linked to normal processes of memory and innate immunity.

In the fungus *Podospora anserina*, the protein HET-S includes a prion-forming domain, which, when it folds into the so-called beta-solenoid conformation, causes the protein to embed in the plasma membrane, where it forms a pore that ultimately results in cell death. One trigger for this folding is interaction with a very similar protein (called HET-s) from a different strain of the same fungus. When the two strains meet, their prion-mediated interaction prevents them from fusing and limits the transmission of pathogens between strains.

**Figure 1:**
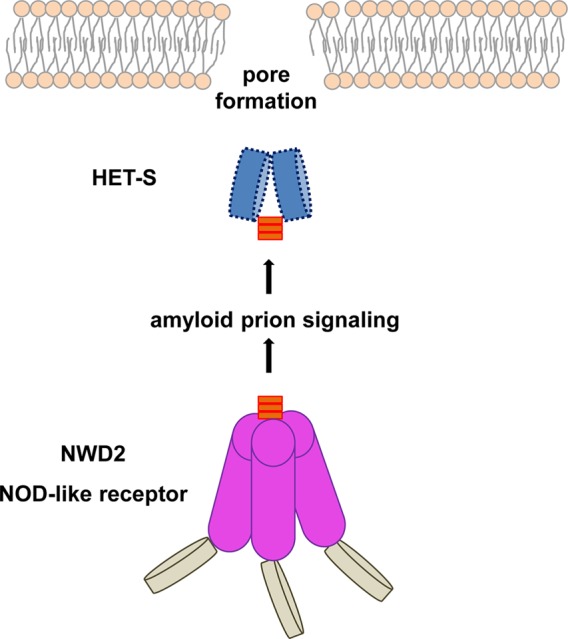
The fungal NOD-like receptor NWD2 controls activation of the HET-S pore-forming protein. The signaling process is based on the formation and transmission of an amyloid prion structure.

But HET-s isn’t the only protein that induces prion folding in HET-S, according to a new study by Asen Daskalov, Sven Saupe, and colleagues in this issue of *PLOS Biology*. They show that the HET-S conformation change can be triggered through interaction with a member of a widely distributed protein family, suggesting that prion-based signal transduction may be more common than currently appreciated (Fig. 1).

Several lines of evidence suggested that the protein, NWD2, might be a HET-S prion partner. The gene sits right next to the gene for HET-S, and the NWD2 protein contains a short beta-solenoid motif of its own. It is a member of the signal transduction ATPases with numerous domains (STAND) family of proteins, which function in innate immunity systems in a wide range of organisms, including humans, in whom APAF-1 induces apoptosis.

NWD2 is a receptor, but its ligand is unknown, so to test whether NWD2 and HET-S interact, the authors constructed chimeric proteins, in which the ligand-binding domain was replaced with homologous regions from related proteins whose ligands are known. Introduction of those ligands induced folding in the prion-forming domain of a HET-S/s protein. Mutation of NWD2 residues that altered the beta-solenoid domain of NWD2 prevented this induction. NWD2’s prion-forming domain alone could induce prion formation in HET-S/s proteins, even without ligands, suggesting an inhibitory role for some part of the protein that was removed. When portions of NWD2 and HET-S were transfected into yeast, which doesn’t contain its own version of these proteins, prion formation was nonetheless induced, indicating that other cellular partners are likely not needed to trigger prion folding in HET-S, as long as NWD2 is present.

The authors note that the HET-S/NWD2 system is found widely in the fungal kingdom, while the HET-S/HET-s incompatibility system is limited to *P*. *anserina*, suggesting that the latter system is an evolutionarily modified version of the former one. Whether the same mechanism is at work in related prion-forming proteins in mammals remains to be seen, but several structural features, including its compactness and its ability to tightly regulate folding, may give this system advantages for signal transduction, including in, but perhaps not limited to, cell defense systems of the immune system.
